# Life-threatening ventricular arrhythmia recognition by nonlinear descriptor

**DOI:** 10.1186/1475-925X-4-6

**Published:** 2005-01-24

**Authors:** Yan Sun, Kap Luk Chan, Shankar Muthu Krishnan

**Affiliations:** 1 Bioinformatics Institute, 138671 Singapore; 2 Biomedical Engineering Research Center, School of Electrical and Electronic Engineering, Nanyang Technological University, 639798 Singapore

## Abstract

**Background:**

Ventricular tachycardia (VT) and ventricular fibrillation (VF) are ventricular cardiac arrhythmia that could be catastrophic and life threatening. Correct and timely detection of VT or VF can save lives.

**Methods:**

In this paper, a multiscale-based non-linear descriptor, the Hurst index, is proposed to characterize the ECG episode, so that VT and VF can be recognized as different from normal sinus rhythm (NSR) in the descriptor domain.

**Results:**

This newly proposed technique was tested using MIT-BIH malignant ventricular arrhythmia database. The relationship between the ECG episode length and the corresponding recognition performance was studied. The experiments demonstrated good performance of the proposed descriptor. An accuracy rate as high as 100% was obtained for VT/VF to be recognized from NSR; for VT and VF to be recognized from each other, the recognition accuracy varies from 84.24% to 100%. In addition, the results were compared favorably against those obtained using Complexity measure.

**Conclusions:**

There is strong potential for using the Hurst index for malignant ventricular arrhythmia recognition in clinical applications.

## Introduction

If a life-threatening ventricular tachycardia (VT) or ventricular fibrillation (VF) is detected promptly, a high energy electrical shock can be delivered to the heart, in an attempt to return the heart to a normal sinus rhythm (NSR). If a normal sinus rhythm is misinterpreted as VT or VF, leading to delivering of an unnecessary shock, it can damage the heart, causing fatal consequences to the patient. Therefore, correct and prompt detection of VT or VF is of great importance. However, the detection of these life-threatening cardiac arrhythmia is difficult because the waveform and frequency distribution of these life-threatening arrhythmia changes with the prolonged duration [1]. Furthermore, practical problems such as poor contact, movement, interference, etc, can produce artifacts that mimic these rhythms [2].

Till now, many linear techniques for VT/VF detection have been developed, such as the probability density function method [3], rate and irregularity analysis [4], analysis of peaks in the short-term autocorrelation function [5], sequential hypothesis testing algorithm [6,7], correlation waveform analysis [8], four fast template matching algorithms [9], VF-filter method [2,10], spectral analysis [1], and time-frequency analysis [11]. However, these methods exhibit disadvantages, some being too difficult to implement and compute for automated external defibrillators (AED's) and implantable cardioverter defibrillators (ICD's), and some only successful in limited cases. For example, the linear techniques [5,11] using the features of amplitude or frequency have shown their limits, since the amplitude of ECG signal decreases as the VF duration increases, and the frequency distribution changes with prolonged VF duration. Therefore, more sophisticated signal processing techniques are needed to fully describe and characterize VT and VF and facilitate the development of new detection schemes with high correct detection rate, or equivalently, with low false-positive and false-negative performance statistics.

Recent studies [12,13] have shown that the cardiac dynamics are complex and non-linear. Even if they could be described by a set of differential equations, they would be of high dimensionality. Normally, each heart beat is initiated by a stimulus from pacemaker cells in the SA node in the right atrium. The activation wave then spreads through the atria to the AV junction. Following activation of the AV junction, the cardiac impulse spreads to the ventricular myocardium through a specialized network, the His-Purkinje system. This branching structure of the conduction system is a self-similar tree with finely scaled details on a microscopic level. The spread of the depolarization wave is represented by the QRS complex in ECG. Spectral analysis of the waveform reveals a broadband of frequencies. To explain the inverse power-law spectrum, West has conjectured that the repetitive branches of the His-Purkinje system represent a fractal set in which each generation of the self-similar tree imposes greater detail onto the system [14]. The effect of the finely branching fractal network is to subtly decorrelate the individual pulses that superpose to form the QRS complex. The distribution in path lengths resulting from the fractal nature of the branches give rise to a distribution of decorrelation time. Some methods developed based on the theory of non-linear dynamics have been highlighted for the analysis of the signals generated from non-linear system [15]. Due to the complex and non-linear dynamical behavior of the cardiac conduction system, non-linear dynamics or non-linear mathematical models are considered to be suitable tools for the analysis of ECG signals. Non-linear techniques have been proven to be major cornerstones for understanding the ECG signals [13,16,17].

Some non-linear techniques [18-20] have been developed for life-threatening ventricular arrhythmia recognition. However, there are still many problems requiring solution. The computational demands for most of the existing algorithms are considerably high and a long ECG episode duration is needed. In order to strike a balance between lower computational burden and reliable recognition performance, a non-linear descriptor, the Hurst index, is proposed as a new tool in this study for recognition of the life-threatening ventricular arrhythmia. The Hurst index is defined in the multiscale domain as a feature to quantify the non-linear dynamical behavior (such as, self-similarity, roughness and irregularity) of the ECG signal for detecting the life-threatening ventricular arrhythmia.

ECG episodes with VT and VF from MIT-BIH malignant arrhythmia database [21] are tested for cardiac abnormality recognition. The data also included some NSR signals to check on the validity of the algorithm. Experimental results are compared with those obtained by a typically used non-linear technique, the Complexity measure, which has been shown to perform well for life-threatening ventricular arrhythmia recognition [20]. In this paper, the complexity measure is Zheng's complexity measure without exception. Detailed description of Zheng's complexity measure technique can be find in [20].

The present paper is organized as follows. Mathematical background on the proposed non-linear descriptor is given in Section. Methodology for the recognition of ventricular arrhythmia is described in Section. Section covers the experimental results and discussions. Lastly, a conclusion of the proposed study is given in Section.

### Multiscale-based non-linear descriptor

Multiscale analysis is a useful framework for many signal processing tasks. Wavelet transform is a good tool for multiscale analysis, which allows the expansion of a signal from the time domain into the time-frequency domain. In this paper, the Hurst index, defined in multiscale space, is proposed for the characterization of ECG episodes.

The Hurst index, *H*, is a single scalar parameter describing the fractal Brownian motion (fBm) model, which is a useful model for nonstationary stochastic self-similar processes with long term dependencies over wide ranges of frequencies [22]. fBm is an extension of the ordinary Brownian motion, and is a zero-mean Gaussian nonstationary stochastic process *B*_*H*_(*t*), *t *∈ ℝ, 0 <*H *< 1, [23]. Self-similarity is inherent to the fBm structure. The fractal dimension *D *is a commonly used parameter for measuring self-similarity. The relationship between the fractal dimension, *D*, and the Hurst index *H *is: *D *= *S *- *H*, where *S *is the topology dimension. For a one-dimensional signal, *S *= 2; for a two-dimensional image, *S *= 3 [24]. The fBm model has following features:

• It is non-stationary, which necessitates some time-dependent analysis.

*E*(*B*_*H*_(*t*)*B*_*H*_(*s*)) = *σ*^2^/2(|*t*|^2^*H *+ |*s*|^2^*H *- |*t *- *s*|^2^*H*)     (1)

where *E*(*·*) represents the expectation operator, *σ *is the standard deviation, *t *is a time variable, *s *is a time lag variable. Based on Equation (1), the variance of fBm, is computed as *var*(*B*_*H*_(*t*)) = *σ*^2^|*t*|^2*H*^.

• It is self-similar, which necessitates some scale-dependent analysis.

{*B*_*H*_(*at*)} ≜ *a*^*H *^*B*_*H*_(*t*), *a *∈ ℝ^+ ^    (2)

where ℝ^+ ^is the set of positive real numbers. ≜ means equality in distribution, which means that the fBm has stationary increments, and the probability properties of the process *B*_*H*_(*t *+ *s*) - *B*_*H*_(*t*) only depend on the lag variable *s*. The scalar index *H *of fBm is related to the complexity and roughness of fBm samples.

Consider a discrete orthogonal wavelet decomposition of a given fBm, *B*_*H*_(*t*).



For any given resolution 2^*J*^, the wavelet mean-square representation of fBm is:



Computing the corresponding wavelet coefficients amounts to evaluating the following approximate coefficients *a*_*j*_[*n*] and detail coefficients *d*_*j*_[*n*]:





where *φ*(*t*) is the corresponding smooth function of wavelet *ψ*(*t*).

Flandrin et al. in [22] have deduced the following theorem: When normalized according to



Wavelet coefficients of fBm give rise to:



where *V*_*ψ*_(*H*) is constant, which depends on both the chosen wavelet and the fBm index *H*. It follows the power-law behavior of the wavelet coefficients' variance:

log_2_(*var*(*d*_*j*_[*n*])) = (2*H *+ 1)*j *+ *constant *    (9)

Therefore, the fBm index *H *(and hence the associated fractal dimension *D *= 2 - *H*) can be easily obtained from the slope of this variance plotted as a function of scale in a log-log plot.

### Life-threatening ventricular arrhythmia recognition by Hurst index

For each testing ECG episode, the following steps are performed:

• Perform wavelet decomposition and computation of its detail coefficients at different scales.

• Compute the Hurst index *H *according to Equation (9).

• Detect the life-threatening ventricular arrhythmia in the feature space of *H*.

In this study, the wavelet used is a quadratic spline wavelet with compact support and one vanishing moment. It is a first derivative of a smooth function [25], whose discrete Fourier transform is:



The low-pass and high-pass filters *L*(*ω*) and *G*(*ω*) are respectively:





The dyadic wavelet transform (WT) of a digital signal *f*(*n*) can be calculated with Mallat's algorithm [26] as follows:





where  is a smoothing operator.  is the wavelet transform of digital signal *f*(*n*). *l*_*k*_|*k *∈ *Z *and *g*_*k*_|*k *∈ *Z *are coefficients of a low-pass filter *L*(*ω*) and a high-pass filter *G*(*ω*), respectively, and, *L*(*ω*) = Σ_*k*∈*Z*_*l*_*k*_*e*^-*ikω*^, *G*(*ω*) = Σ_*k*∈*Zgk*_*e*^-*ikω*^. Based on the frequency analysis of the ECG characteristic waves [27], scale 2^*j *^(*j *= 1 to 4) are selected. For each experimental episode, its wavelet transform coefficient sets *d*_1_, *d*_2_, *d*_3 _and *d*_4 _corresponding to different scales 2^1^, 2^2^, 2^3^, 2^4 ^are computed. The Hurst index *H *is then computed according to Equation (9). Smaller Hurst index corresponds to larger fractal dimension and more irregular signal.

### Comparative Experimental Results and Discussions

#### Description of the test data

**Figure 1:**
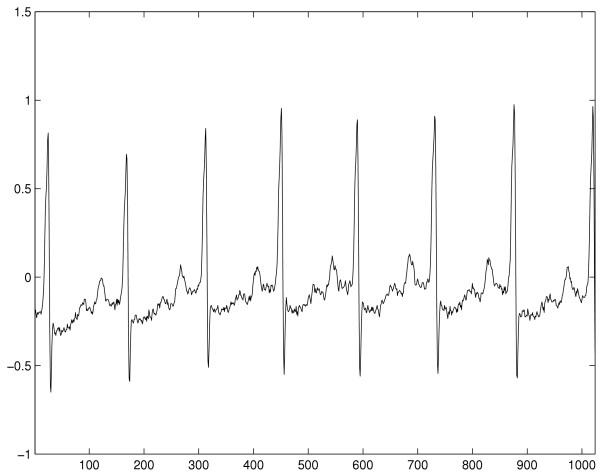
Typical life-threatening ECG waveform of NSR

**Figure 2:**
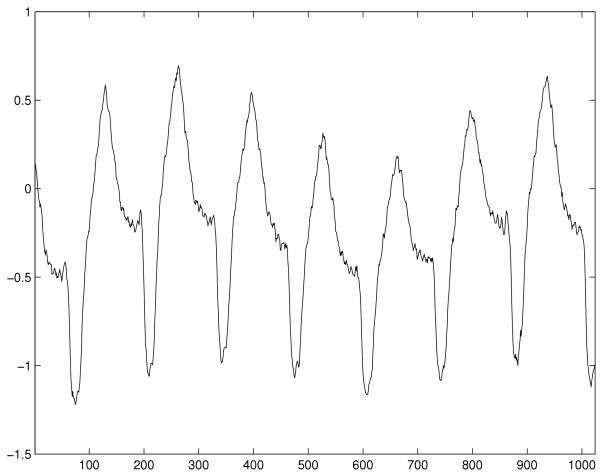
Typical life-threatening ECG waveform of VT

**Figure 3:**
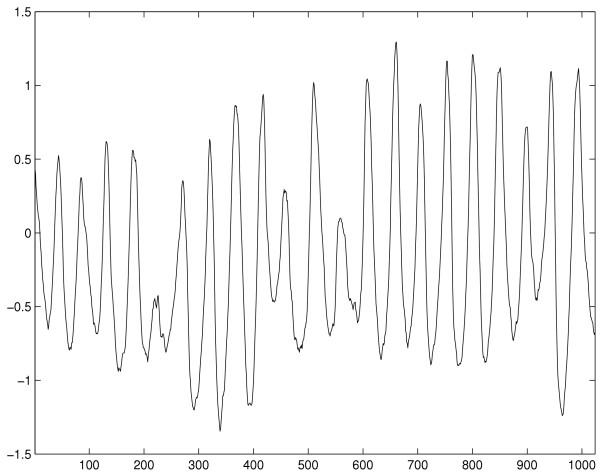
Typical life-threatening ECG waveform of VF

The database used in this study is the MIT-BIH malignant ventricular arrhythmia database [21] with a sample frequency of 250 *Hz*. Typical waveforms of VT and VF as well as NSR are shown in Figure 1 to 3. Selected ECG episodes with different lengths are tested for evaluating the performance of the life-threatening ventricular arrhythmia recognition using the Hurst index. Each ECG episode is characterized by the Hurst index *H*, computed by Equation (9). The statistical distribution of the Hurst indexes for characterizing different types of episodes is studied so that VT and VF can be recognized in the feature domain of the Hurst index. Recognition performance is measured by *Sensitivity *(*SE*), *Specificity *(*SP*) and *Accuracy *(*ACR*). They are defined as: *Sensitivity *= ; *Specificity *= ; *Accuracy *= . Where *TP *is true positive, the abnormal case being correctly recognized as abnormal one; *FN *is false negative, the abnormal case being wrongly recognized as normal one; *TN *is true negative, the normal case being correctly recognized as normal one; and *FP *is false positive, the normal case being wrongly recognized as abnormal one. Lastly, results are compared with that of Complexity measure technique.

In this study, about 5076 ECG episodes are tested for performance evaluation of life-threatening ventricular arrhythmia recognition using the proposed Hurst index. Among them, 2588 cases are NSR episodes, 1390 cases are VT episodes, and 1098 are VF episodes. In order to explore the effect of the time series lengths on the recognition performance using the proposed Hurst index, analyzing was conducted using different lengths of ECG episodes from 1 *sec *to 5.5 *sec *with a difference of 0.5 *sec*. For each length, the whole dataset was randomly divided into two equal parts for training and testing, respectively. From a clinical point of view, it is essential to recognize and diagnose malignant ventricular arrhythmia as soon as possible. This calls for detection with as short a length of the time series as possible.

**Figure 4:**
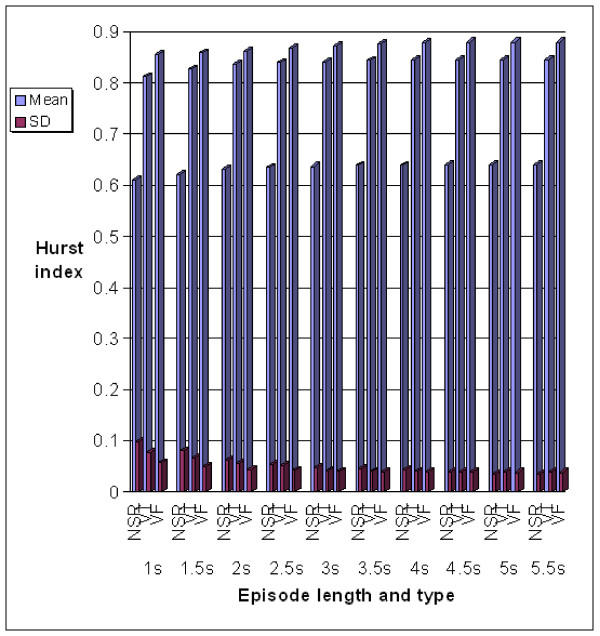
The mean and standard deviation values for characterizing NSR, VT and VF episodes using the Hurst index

**Figure 5:**
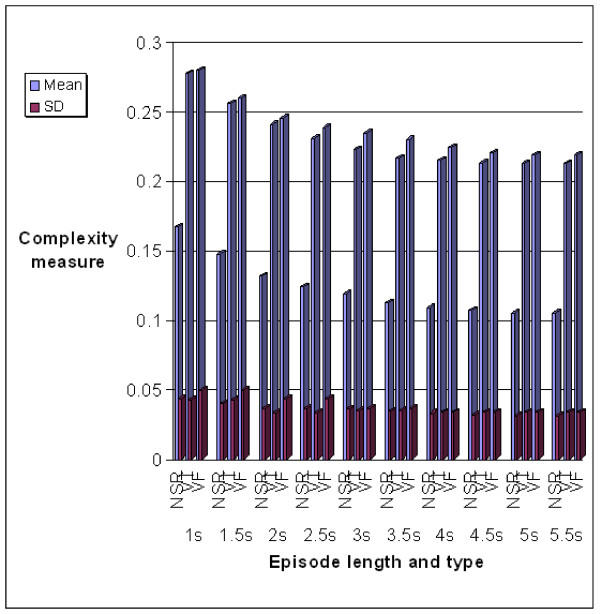
The mean and standard deviation values for characterizing NSR, VT and VF episodes using the Complexity measure

The statistical results, viz, the means and standard deviations for characterizing NSR, VT and VF episodes using the Hurst index are given in Table 1. As a comparison, the results by the complexity measure technique, are given in Table 2. Graphical descriptions of the results listed in Tables 1 and 2 are shown in Figure 4 and 5 respectively.

**Table 1:** Statistical results of Hurst index for episode characterization

Episode Length	Hurst index					
	
	NSR		VT		VF	
	
	Mean	SD	Mean	SD	Mean	SD
1 sec	0.6099	0.0981	0.8117	0.0775	0.8567	0.0579
1.5 sec	0.6206	0.0805	0.8269	0.0671	0.8597	0.0501
2 sec	0.6317	0.0619	0.8373	0.0558	0.8618	0.0438
2.5 sec	0.6349	0.0549	0.8398	0.0509	0.8682	0.0419
3 sec	0.6389	0.0458	0.8445	0.0409	0.8766	0.0399
3.5 sec	0.6389	0.0458	0.8445	0.0409	0.8766	0.0399
4 sec	0.6395	0.0436	0.8452	0.0403	0.8794	0.0395
4.5 sec	0.6398	0.04	0.8455	0.0397	0.8797	0.0392
5 sec	0.6399	0.035	0.8458	0.0391	0.8799	0.0387
5.5 sec	0.6399	0.035	0.8458	0.0388	0.8799	0.0386

**Table 2:** Statistical results of Hurst index for episode characterization

Episode Length	Complexity measure					
	
	NSR		VT		VF	
	
	Mean	SD	Mean	SD	Mean	SD
1 sec	0.1674	0.0433	0.2775	0.0428	0.2798	0.0498
1.5 sec	0.1476	0.0403	0.2562	0.0428	0.2601	0.0498
2 sec	0.1319	0.037	0.2413	0.0335	0.2454	0.0432
2.5 sec	0.1245	0.0366	0.2311	0.0335	0.239	0.0432
3 sec	0.1192	0.0363	0.2229	0.0349	0.2351	0.037
3.5 sec	0.1129	0.0348	0.2168	0.0349	0.2298	0.037
4 sec	0.1095	0.0332	0.2149	0.0342	0.2242	0.0343
4.5 sec	0.1071	0.0321	0.2136	0.0342	0.2205	0.0343
5 sec	0.1056	0.0315	0.2129	0.0342	0.2187	0.0341
5.5 sec	0.1056	0.0313	0.2129	0.0339	0.2187	0.0341

From the results shown in Figure 4 and 5, the following observation can be made.

• As the episode length increases, the mean of Hurst index for every type of rhythm basically increases and tends to approach a relatively stable value, while the standard deviation decreases gradually.

• For a particular episode length, from NSR to VT then to VF, the corresponding Hurst index increases gradually. The increase from NSR to VT is more than the increase from VT to VF.

• As the episode length increases, the mean of Complexity measure for every type of rhythm basically decreases and tends to approach a relatively stable value, while the standard deviation decreases gradually.

• For a particular episode length, from NSR to VT then to VF, both the Hurst index and the Complexity measure increase gradually, in which, the increase from NSR to VF is far more than the increase from VT to VF.

• The mean values of Hurst index vary slower than those of Complexity measure as the episode length increases from 1 *sec *to 5.5 *sec*. It is concluded that the Hurst index is more stable than the Complexity measure with respect to episode lengths.

Using the Hurst index for VT or VF recognition from NSR with different episode lengths, there is no false detection, meaning that the VT/VF can be totally correctly recognized from NSR without exception. For the Complexity measure, when the length of ECG episode is longer than 1 *sec*, it has as good performance as the Hurst index; when the length of the ECG episode is 1 *sec*, there is 6 false negatives and 27 false positives; when the length of the ECG episode is 1.5 *sec*, there is 1 false negatives and 5 false positives. The statistical values of *SE*, *SP *and *ACR *for VT/VF recognition from NSR using the Hurst index are all 100%. Hence, the Hurst index can be used to detect VT and VT earlier.

As for VF differentiation from VT, the statistical values of *SE*, *SP *and *ACR *for different episode lengths using the Hurst index and the Complexity measure, are shown in Table 3. The computational time of the Hurst index and the Complexity measure for different ECG episode length are presented in Table 4. From Table 3, the following conclusions can be obtained:

**Table 3:** Statistical values of *SE*, *SP *and *ACR *for VF differentiation from VT

Episode Length	Hurst index			Complexity measure		
	
	SE	SP	ACR	SE	SP	ACR
1 sec	0.8351	0.8482	0.8424	0.8242	0.8302	0.8275
1.5 sec	0.8780	0.8698	0.8734	0.8689	0.8597	0.8637
2 sec	0.9080	0.8834	0.8942	0.9007	0.8798	0.8890
2.5 sec	0.9408	0.9158	0.9268	0.9381	0.9194	0.9277
3 sec	0.9608	0.9439	0.9513	0.9654	0.9489	0.9562
3.5 sec	0.9754	0.9669	0.9707	0.9818	0.9734	0.9771
4 sec	0.9854	0.9849	0.9851	0.9918	0.9885	0.9899
4.5 sec	0.9936	0.9914	0.9924	1	0.9986	0.9992
5 sec	1	0.9978	0.9988	1	1	1
5.5 sec	1	1	1	1	1	1

**Table 4:** Computation time comparison in seconds

Length of episode	Hurst index	Complexity measure	Length of episode	Hurst index	Complexity measure
1 sec	0.0546	0.0654	1.5 sec	0.0697	0.0824
2 sec	0.0794	0.1143	2.5 sec	0.0933	0.1538
3 sec	0.1168	0.2176	3.5 sec	0.1401	0.2991
4 sec	0.1885	0.4003	4.5 sec	0.2407	0.609
5 sec	0.2803	0.6833	5.5 sec	0.3122	0.7792

• The performance on differentiating VT and VF is worse than the performance of VT/VF recognition from NSR, for both the Hurst index and the Complexity measure.

• The recognition performance by either descriptors improves as the length of ECG episode increases.

• When the length of ECG episode is less than or equal to 2 *sec*, the recognition performance for the Hurst index is better. When the length of ECG episode is longer than 2 *sec *and less than 5 *sec*, the recognition performance for the Complexity measure is better. When the length of ECG episode is longer than 5 *sec*, VT and VF can be 100% differentiated with either descriptor, the recognition performance for both descriptors are same.

According to Table 4, the computational time for the Hurst index is less than that for the Complexity measure. These two algorithms are programmed using MATLAB 5.3 running on a SUN SPARC-333*MHz *workstation. The computational burden for the Hurst index is *O*(*N *log_2 _*N*), while the computational burden for the complexity is *O*(*N*^2^), where *N *is the length of ECG episode. It is noted that with more powerful computer programming in C, the computational speed will be further improved.

Time is an important factor for saving lives in clinical situations, therefore, algorithm with less computational burden is obviously preferred. In addition, using short ECG episode length is preferred for earlier detection of arrhythmia (such as VT/VF). Based on the experimental results, it is observed that the Hurst index has a better potential for clinical adaptation than the Complexity measure.

## Conclusions

In this paper, a new technique based on multiscale analysis and non-linear dynamics was presented for VT and VF recognition. Hurst index defined across multiscale was proposed for characterizing ECG episode so that life-threatening arrhythmia can be recognized. Furthermore, upon applying to the MIT-BIH malignant ventricular arrhythmia database, the performance for malignant arrhythmia recognition using Hurst index was compared with that using Zheng's complexity measure. The Hurst index requires less computation and is more reliable in detecting VT and VF with short ECG episode. There is strong potential for using the Hurst index for malignant ventricular arrhythmia recognition in clinical applications.

## Authors' contributions

SY conceived the study, performed data analysis and drafted the manuscript. CKL and KSM guided the study, helped the analysis and interpretation of the results, and critically reviewed the manuscript. All authors read and approved the final script.

## References

[B1] Barro S, Ruiz R, Cabello D, Mira J (1989). Algorithmic sequential decision-making in the frequency domain for life threatening ventricular arrhythmias and imitative artifacts: a diagnostic system. J Biomed Eng.

[B2] Clayton RH, Murray A, Campbell RW (1993). Comparison of four techniques for recognition of ventricular fibrillation from the surface ECG. Med Biol Eng Comput.

[B3] Langer A, Heilman MS, Mower MM (1976). Considerations in the development of the automatic implantable defibrillator. Medical Instrumentation.

[B4] Ripley KL, Bump TE, Arzbaecher RC (1989). Evaluation of techniques for recognition of ventricular arrhythmias by implanted devices. IEEE Transactions on Biomedical Engineering.

[B5] Chen S, Thakor NV, Mover MM (1987). Ventricular fibrillation detection by a regression test on the autocorrelation function. Med Biol Eng Comput.

[B6] Thakor NV, Natarajan A, Tomselli G (1994). Multiway sequential hypothesis testing for tachyarrhythmia discrimination. IEEE Transactions on Biomedical Engineering.

[B7] Chen SW, Clarkson PW, Fan Q (1996). A robust detection algorithm for cardiac arrhythmia classification. IEEE Transactions on Biomedical Engineering.

[B8] Lin D, Jenkins JM, DiCarlo LA, MacDonald RS (1988). Arrhythmia diagnosis using morphology and timing from atrial and ventricular leads. Computers in Cardiology.

[B9] Throne RD, Jenkins JM, DiCarlo LA (1991). A comparison of four new time-domain techniques for discriminating monomorphic ventricular tachycardia from sinus rhythm using ventricular waveform morphology. IEEE Transactions on Biomedical Engineering.

[B10] Kuo S, Dillman R (1978). Computer detection of ventricular fibrillation. Comput Cardiol.

[B11] Afonso VX, Tompkoins WJ (1995). Detecting ventricular fibrillation: selecting the appropriate time-frequency analysis tool for the application. IEEE Engineering in Medicine and Biology.

[B12] Dirk H, Bernd P, Hanspeter H, Ulrich Z (1998). Non-linear coordination of cardiovascular automatic control. IEEE Engineering in Medicine and Biology.

[B13] Seidel H, Herzel H (1998). Investigating the dynamics of atrioventricular delay. IEEE Engineering in Medicine and Biology.

[B14] West BJ (1990). Fractal Physiology and Chaos in Medicine. World Scientific, Singapore.

[B15] Abarbanel HI (1996). Analysis of Observed Chaotic Data.

[B16] Fojt O, Holcik J (1998). Applying non-linear dynamics to ECG signal processing. IEEE Engineering in Medicine and Biology.

[B17] Cohe ME, Hudson DL, Deedwania PC (1996). Applying continuous chaotic modeling to cardiac signal analysis. IEEE Engineering in Medicine and Biology.

[B18] Ravelli F, Antolini R (1992). Complex dynamics underlying the human electrocardiogram. Biol Cybern.

[B19] Jenkins JM, Caswell SA (1996). Detection algorithms in implantable cardioverter defibrillators. Proc IEEE.

[B20] Zheng XS, Zhu YS, Thakor NV, Wang ZZ (1999). Detecting ventricular tachycardia and fibrillation by complexity measure. IEEE Transactions on Biomedical Engineering.

[B21] MIT-BIH arrhythmia database. http://www.physionet.org/physiobank/database/mitdb/.

[B22] Flandrin P (1992). Wavelet analysis and synthesis of fractal Brownian motion. IEEE Transactions Information Theory.

[B23] Mandelbrot BB, VanNess JW (1968). Fractal Brownian motions, fractional noises and applications. SIAM review.

[B24] Falconer K (1990). Fractal Geometry.

[B25] Mallat S (1992). Characterization of signals from multiscale edges. IEEE Transactions on Pattern Analysis and Machine Intelligence.

[B26] Mallat S (1991). Zero-crossing of a wavelet transform. IEEE Transactions on Information Theory.

[B27] Li C, Zheng C, Tai CF (1995). Detection of ECG characteristic points using wavelet transforms. IEEE Transactions on Biomedical Engineering.

